# Wearable Intracranial Pressure Monitoring Sensor for Infants

**DOI:** 10.3390/bios11070213

**Published:** 2021-06-29

**Authors:** Baoyue Zhang, Ziyi Huang, Huixue Song, Hyun Soo Kim, Jaewon Park

**Affiliations:** 1 School of Microelectronics, Southern University of Science and Technology, Shenzhen 518055, China; by51588986@163.com (B.Z.); 11510386@mail.sustech.edu.cn (Z.H.); songhx@mail.sustech.edu.cn (H.S.); 2 Department of Electronic Engineering, Kwangwoon University, Seoul 01897, Korea

**Keywords:** wearable sensor, intracranial pressure sensor, liquid metal, freeze casting

## Abstract

Monitoring of intracranial pressure (ICP) is important for patients at risk of raised ICP, which may indicate developing diseases in brains that can lead to brain damage or even death. Monitoring ICP can be invaluable in the management of patients suffering from brain injury or hydrocephalus. To date, invasive measurements are still the standard method for monitoring ICP; however, these methods can not only cause bleeding or infection but are also very inconvenient to use, particularly for infants. Currently, none of the non-invasive methods can provide sufficient accuracy and ease of use while allowing continuous monitoring in routine clinical use at low cost. Here, we have developed a wearable, non-invasive ICP sensor that can be used like a band-aid. For the fabrication of the ICP sensor, a novel freeze casting method was developed to encapsulate the liquid metal microstructures within thin and flexible polymers. The final thickness of the ICP sensor demonstrated is 500 µm and can be further reduced. Three different designs of ICP sensors were tested under various pressure actuation conditions as well as different temperature environments, where the measured pressure changes were stable with the largest stability coefficient of variation being only CV = 0.0206. In addition, the sensor output values showed an extremely high linear correlation (R^2^ > 0.9990) with the applied pressures.

## 1. Introduction

**Figure 1:**
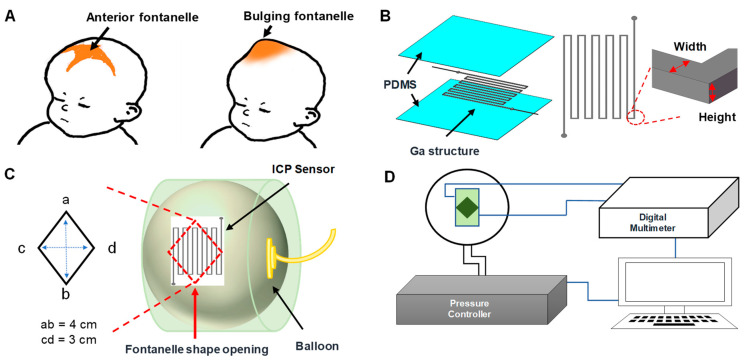
(**A**) An infant with a normal fontanelle (left) and a bulging fontanelle (right). (**B**) An illustration showing the design of the ICP sensor. (**C**) An image of the developed balloon-in-box fontanelle model (inset: opening area of the fontanelle model). (**D**) A schematic illustration of the overall experimental setup.

It has been proposed that the anterior fontanelle, the soft area of the unformed skull on a new-born baby, can be used as a site for detecting the increased intracranial pressure (ICP) of infants [1,2]. ICP levels for normal infants are 5–10 mbar and the ICP beyond these values may be the clinical expression of several intracranial pathologies [3,4,5,6,7]. In addition, increased ICP of infants not only interferes with normal brain development but also causes the fontanelle to bulge out significantly (Figure 1A). Unfortunately, monitoring the ICP of infants is not easy as commonly used conventional systems are either too expensive (i.e., non-invasive methods) or designed for adult patients and too big (i.e., invasive methods) for infants, which can bring larger pain and easily cause infections to infants having weaker physical conditions and immune systems. In such circumstances, an ICP monitoring sensor that can be easily used for infants in a non-invasive manner may be of a significant value.

Various methods have been developed to measure and monitor the ICP, which can be categorized into two different approaches; invasive and non-invasive. For the invasive methods, lumbar puncture is the simplest and longest-standing technique [8]. However, this indirect method can be inaccurate and involves a painful process. The ventricular catheterization method [9,10], considered as the gold standard for the ICP measurement, has several critical weaknesses; it is vulnerable to infection and is not suitable for long-term ICP monitoring. Another invasive method includes inserting microtransducers in the brain parenchyma, typically placed in the right frontal region, yet this method requires a surgical process and also suffers from brain swelling issues and baseline drift of the measurements over time [11]. In terms of non-invasive approaches, transcranial Doppler (TD) [12,13,14], tympanic membrane displacement (TMD) [15,16], and magnetic resonance imaging (MRI) [17,18] are utilized. The TD technique applies ultrasound to measure the blood flow velocity in the middle cerebral artery. Although convenient, as the ICP increases, the TD measurement results fluctuate, which induces a higher magnitude of deviation. For the TMD method, the variability between subjects were too large, and thus, its clinical application was not successful. MRI is a very powerful tool that can detect the ICP in a non-invasive manner; however, the method requires an expensive and bulky system and is not suitable for real time monitoring of the ICP. Overall, all of the invasive techniques hold risk of complications such as hemorrhage, infection, and system malfunction, while many non-invasive methods either lack accuracy or involve high expenses.

With the development of microfabrication technologies, various types of miniaturized silicon-based pressure sensors (e.g., capacitive, piezoelectric, optic) have been reported and investigated [19,20,21,22]. Although these pressure sensors have advantages of high sensitivity, linearity, and accuracy, their use in biomedical applications, particularly for wearable types, have been limited largely due to their low stress strength. To overcome such limitations, flexible sensors have become of interest to both academics and industries [23,24,25,26,27,28,29,30]. Liquid metals are a special family of materials that simultaneously possess the properties of both metals and liquids [31,32,33]. Particularly, room-temperature liquid metals refer to these metals whose melting points are lower than 30 °C. For example, as ‘liquid,’ gallium (Ga), with a low melting point (29.8 °C), has a low viscosity and a high surface tension, while as ‘metal,’ it has high thermal and electrical conductivities. Owing to its liquid phase nature, Ga can contact with any shape of objects and maintain excellent electrical properties even when the substrate or encapsulating films change their form or shape. Adopting Ga for flexible devices can achieve higher flexibility and extensibility of devices, as well as high stability and reliability under deformation/stress working conditions. Additionally, unlike some liquid metals (e.g., mercury), Ga is non-toxic and is being widely used in various fields, including biomedical applications [34,35,36,37,38,39,40,41,42,43].

In this research, we introduce a Ga-based wearable pressure sensor for non-invasive ICP monitoring of infants, which can be easily used like a band-aid. Infants’ ICP change is monitored by measuring the electrical resistance change of the liquid metal-filled microchannel deformed by the inflation. In addition, a novel fabrication method for encapsulating the Ga structure within a very thin, flexible, and stretchable polymer (i.e., PDMS) was developed, since previously reported liquid metal-based pressure sensors had thick design configurations and were also not suitable for making readily inflatable sensors. As a proof-of-concept study, a balloon-in-box fontanelle model system was developed and utilized to validate and characterize the performance of the developed ICP sensor. The developed sensor can be fabricated at significantly lower cost compared to any existing methods and can be further improved by integrating it with a wireless communication module for convenient clinical applications or point-of-care system. In addition, it can even be used for analysis of professional athletes’ performance, movement development, or motion capture applications.

## 2. Materials and Methods

### 2.1. ICP Sensor Design

Figure 1B shows the design of the developed ICP sensor. The sensor is configured by encapsulating a thin layer of the serpentine-shaped Ga structure (height: 100 µm or 300 µm) between two PDMS layers. The sensing mechanism of the developed sensor is based on Ohm’s law, R = ρL /A, where ρ is the resistivity, L is the length, and A is the cross-sectional area of the liquid metal structure. The electrical resistance of the ICP sensor is proportional to the length of the Ga structure and inversely proportional to the cross-sectional area of the Ga structure. As the developed ICP sensor is very thin and flexible (thickness: 500 µm), the sensor can be easily deformed when exposed to any external force. The deformation directly affects and modifies the encapsulated Ga structure, resulting in its electrical resistance change. In other words, the deformation of the sensor caused by the ICP variation will change the electrical resistance of the sensor, which can be directly measured in real time. To investigate the effect of the liquid metal design on the sensitivity and the stability, Ga structures with three different dimensions were designed and tested (Table 1).

### 2.2. Artificial Fontanelle Model

In order to evaluate the performance of the ICP sensor, a balloon-in-box fontanelle model was developed. The cylindrical fontanelle model (diameter: 120 mm, height: 95 mm) has a diamond-shaped opening (3 × 4 cm^2^) to mimic the opening of anterior fontanelle of infants, and encloses a balloon (Elite, Durex^®^, Parsippany, NJ, USA) connected to a computer-controlled pressure pump (OB1, Elveflow, Paris, France) (Figure 1C,D). The typical opening area of the anterior fontanelle in normal infants is known to be 2 × 3 cm^2^ [44,45], but considering that the opening area of the bulging fontanelle for ill infants is usually larger than that of healthy infants [46,47,48,49], we have designed the model to have a slightly larger opening (Figure 1C). Pressures from 0 mbar to 220 mbar were applied to the balloon for testing the operational pressure range of the developed fontanelle model. The overall system setup is shown in Figure 1D.

### 2.3. ICP Sensor Fabrication

**Figure 2:**
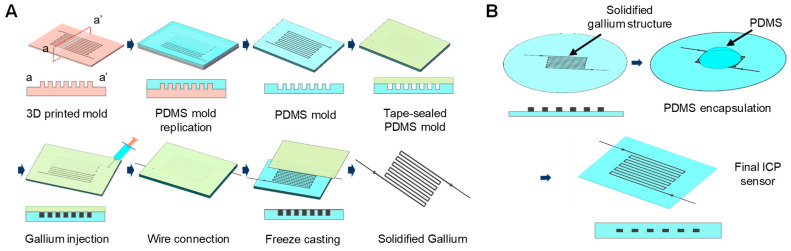
(**A**) Overall freeze casting fabrication process of the Ga structure. (**B**) Encapsulation of the solidified Ga structure with thin PDMS layers.

A novel freeze casting method was developed to easily fabricate a thin liquid metal structure. The overall freeze casting fabrication process of the Ga structure is illustrated in Figure 2A. First, a 3D printed mold for replicating a PDMS mold to freeze and cast the Ga structure was prepared. The mold was designed using the 3D CAD software (SolidWorks Corporation, Waltham, MA, USA) and was printed with a high temperature molding resin (HTM140 V2, EnvisionTEC Inc., Gladbeck, Germany) using a stereolithography type 3D printer (PERFACTORY^®^, EnvisionTEC Inc., Gladbeck, Germany). The printed mold was rinsed with isopropyl alcohol (IPA) for 5 min and dried with N_2_ gas, followed by additional exposure to UV lights for 500 s (OmniCure, Excelitas, Mississauga, ON, Canada). The 3D printed mold was then coated with trichloro (1H, 1H, 2H, 2H-tridecafluoro-n-octyl)silane (T2577, TCI, Tokyo, Japan) to facilitate the detaching process and the uncured PDMS (elastomer base and curing agent mixed at 10:1 ratio) was poured onto the 3D printed mold, followed by curing in an 80 °C oven for 4 h. The replicated PDMS mold was sealed with a Kapton tape^®^ (Misumi Group Inc., Tokyo, Japan), and liquid phase gallium (Ga) was injected with a syringe. The Ga was heated to 80 °C prior to the injection to ensure and maintain its liquid phase during the process. Ga was chosen as the liquid metal material for the ICP sensor due to its higher freezing temperature (melting point = 29.8 °C). After Ga injection, two copper wires were inserted into each end of the serpentine structure to make electrical contacts, followed by solidifying it inside a −20 °C freezer for 15 min. Upon solidification, the sealing tape was removed and the solidified serpentine-shaped Ga structure was peeled off from the PDMS mold. As shown in Figure 2B, the Ga structure was then immediately placed on top of a PDMS membrane prepared by a spin-coating process and additional uncured PDMS was poured for encapsulation, followed by curing at 80 °C for 4 h to obtain the final ICP sensor (thickness: 500 µm).

### 2.4. ICP Sensor Characterization

The ICP sensor was attached to the opening of the balloon-in-box fontanelle model and was connected to a digital multimeter (34461A, Keysight, Wokingham, UK). Change of the electrical resistance of the ICP sensor by the fontanelle inflation was measured and recorded with the connected computer. The sensitivity of the sensors was analyzed by measuring how much the electrical resistances change in accordance to the applied pressures under two different conditions (ΔP = 50 mbar and 100 mbar). The sensitivity was defined as the ratio of the resistance change to the initial electrical resistance value (Sensitivity = ΔR/R_0_, ΔR is the variation of the electrical resistance and R_0_ is the initial electrical resistance). The performance of the sensor (i.e., stability, reliability, linearity) was characterized by applying cyclic incremental pressures and periodic pressures to the fontanelle model. A cyclic pressure with 10 mbar increment (ΔP = 0 mbar to 100 mbar) and a periodic pressure (ΔP = 0 mbar and 100 mbar), both of them with 180 s intervals, were applied over 30 min for three different ICP sensor designs and their electrical resistance changes were measured. All data shown were measured in real time from at least three independent experiments (*n* ≥ 3).

## 3. Results and Discussions

### 3.1. Fabricated ICP Sensor

**Figure 3:**
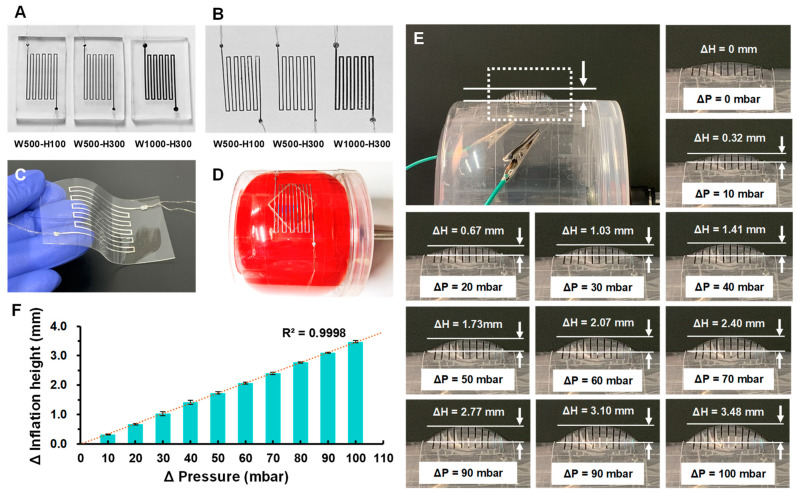
Solidified Ga structures of three different designs with contact wires (**A**) in the PDMS mold after incubating in a −20 °C freezer and removing the sealing tape and (**B**) after peeling off from the PDMS molds. Image of (**C**) the final ICP sensor and (**D**) the artificial fontanelle model assembled with the ICP sensor. A red balloon was used for visualization purposes. (**E**) A balloon-in-box fontanelle model assembled with an ICP sensor (W500-H300), showing different inflation heights at different pressure levels. (**F**) Correlation between the applied pressure and the inflation height of the fontanelle model (R^2^ = 0.9998).

Figure 3A shows the solidified serpentine-shaped Ga structure in the PDMS mold after the freezing step and removing the sealing tape. During the optimization of the developed freeze casting method, we have found that the thickness of the PDMS mold plays an important role in obtaining the solidified Ga structures. When PDMS molds were too thick, the structures could not be easily peeled off due to the stiffness of the mold and this even led the structures to be damaged during the peeling-off process. On the other hand, if the PDMS molds were too thin, the structures were easily deformed during the peeling-off process, where maintaining their shapes was difficult. The optimal thickness of the PDMS mold was found to be 3–4 mm and the Ga structures of all three designs were intactly peeled off from the PDMS mold (Figure 3B). Figure 3C,D show the final ICP sensor (5 cm × 7 cm × 500 µm), where the Ga structure was encapsulated between two thin PDMS layers. The PDMS mold could be used multiple times and no noticeable difference or defects were observed even after replicating the Ga structures 20 times from a single PDMS mold.

The most commonly used method to fill PDMS microchannels with liquid metals is to assemble the PDMS layer either on a glass slide or another PDMS layer, and then directly inject the liquid metal using a syringe with a tubing connection. Unfortunately, for very thin PDMS devices, the direct injection is extremely difficult and time-consuming, and even if the injection is possible, injection interfaces can be easily perforated as microchannels often either collapse or burst during the injection process. The novel freeze casting fabrication method, developed in this paper, allows for easy and simple encapsulation of liquid metal components within thin polymer layers (e.g., PDMS) and successfully demonstrates the possibility of employing liquid metal-based microstructures to fabricate thin and flexible devices for other various applications. In addition, the developed method enables the ICP sensor to be manufactured at low cost without any expensive equipment, making it even disposable after each use, which is a significant advantage for medical devices, eliminating potential contamination issues that comes from reusing the devices. There are multiple techniques currently used to monitor ICP, yet many of them are either invasive, not patient-friendly, or expensive. The cost for placing external ventricular drainage for example, which is considered to be the gold standard, is around US $200 in material cost, it becomes more expensive when using microtransducers that can easily go up to several thousand dollars [50,51]. The proposed non-invasive wearable ICP sensor can be made at extremely low cost, no more than US $5 per sensor unit itself.

### 3.2. Fontanelle Model Validation

The sensitivity of the balloon inflation, in other words, the expansion in accordance to the applied pressure, depends on the material properties of the balloon. We have tested balloons with different materials and thicknesses and chose a 65 µm thick natural rubber latex balloon for this study, as it had sufficient sensitivity with proper durability. The operational pressure range test results showed that 30 mbar was the minimum pressure required for inflating the balloon to make contact with the sensor, and 220 mbar was the maximum pressure the balloon could withstand before bursting. Operation of the balloon-in-box fontanelle model was characterized by measuring the inflation height of the attached ICP sensor (W500-H300) under various applied pressure conditions, ranging from 30 mbar (ΔP = 0 mbar) to 130 mbar (ΔP = 100 mbar) with a 10-mbar increment. The inflation height was measured from photographic images taken at corresponding pressure levels. Two lines were drawn at the top of the inflated sensor and at the opening area of the fontanelle. Distance between two lines was measured in pixels and then converted to millimeters (Figure 3E). As shown in Figure 3E,F, the attached ICP sensor inflated from 0 mm to 3.48 mm as the applied pressure increased to ΔP =100 mbar. Within the tested pressure range, the proposed model showed a high linearity (R^2^ = 0.9998) between the applied pressure and the inflation height. The model having the linear characteristic can be beneficial especially when characterizing the pressure sensors, since the sensitivity or the sensor operating ranges can be more easily and accurately analyzed, ruling out the influence from the model. Depending on needs, the inflation height of the model, in other words, the slope in Figure 3F, can be adjusted by using balloons with different properties (e.g., thickness and elasticity).

### 3.3. ICP Sensor Design Analysis

**Figure 4:**
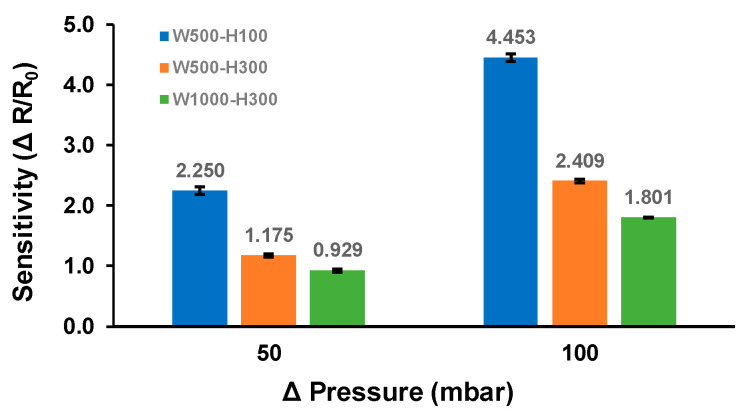
Comparison of the ICP sensor sensitivity among different designs (*n* = 3).

The average sensitivity of the different sensor designs is shown in Figure 4; 2.250 ± 0.065 (W500-H100), 1.175 ± 0.028 (W1000-H300), and 0.929 ± 0.030 (W500-H300) at ΔP = 50 mbar; 4.453 ± 0.060 (W500-H100), 2.409 ± 0.026 (W1000-H300), and 1.801 ± 0.008 (W500-H300) at ΔP = 100 mbar. Although the absolute value of the electrical resistance changes was not large (from a few tens to hundreds of mΩ), they showed very consistent and repeatable measurements for all tested designs. The analysis showed that the sensitivity of the ICP sensor is strongly dependent on the dimensions of the liquid metal structures. The sensitivity of the W500-H100 design was 91.5% and 142.2% higher at 50 mbar and was 84.8% and 147.3% higher at 100 mbar than those of W500-H300 and W1000-H300 designs, respectively. When comparing the ΔR between W500-H100 and W500-H300 (same width but different height), the design with lower height displayed approximately 3.2 times higher change regardless of the pressure levels, which is close to a theoretical estimation of 3. On the other hand, for ΔR between W500-H300 and W1000-H300 (same height but different width), it was only 1.6 times and 1.7 times higher at ΔP = 50 mbar and ΔP = 100 mbar, respectively, which are less than a theoretical estimation of 2. It is expected that the lower aspect ratio liquid metal design resulted in more vertical deformation of the Ga structure during the fontanelle model inflation, making the width a more dominant factor than the height for the sensitivity of the proposed design.

### 3.4. Performance of the ICP Sensor

**Figure 5:**
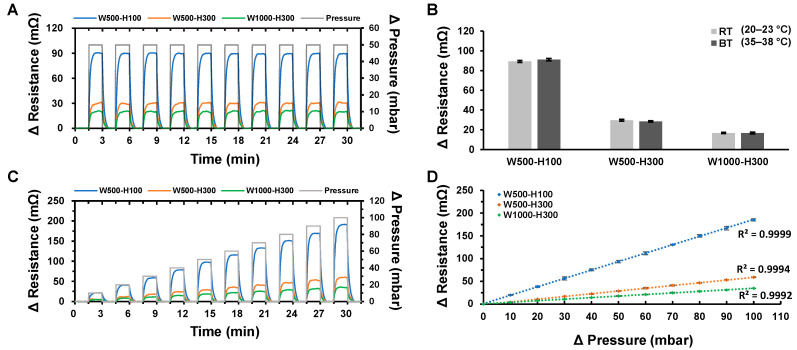
(**A**) Change of the electrical resistance in accordance to the periodic pressure changes (*n* = 3). (**B**) Comparison of the ΔR measurements at two different temperatures (RT = 23–25 °C, BT = 35–38 °C). (**C**) Change of the electrical resistance in accordance to the incremental pressure changes (*n* = 3). (**D**) Graph showing the linearity between the applied pressure and the measured electrical resistance (*n* = 3).

Stability/reliability of the developed ICP sensor was investigated by applying a periodic pressure of ΔP = 0 mbar and ΔP = 50 mbar with 180 s intervals. As shown in Figure 5A, the variation of the electrical resistance values was very consistent from measurements to measurements for all designs. The average ΔR values are 89.51 ± 0.35 mΩ (W500-H100), 30.04 ± 0.37 mΩ (W500-H300), and 20.25 ± 0.42 mΩ (W1000-H300), with CV being only 0.0039, 0.0124, and 0.0206, respectively. The stability/reliability of the ICP sensor was further investigated by analyzing ΔR at two different temperature conditions; room temperature (RT = 23–25 °C) and body temperature (BT = 35–38 °C). When ΔP of 100 mbar was applied, differences of ΔR between RT and BT were insignificant, with ΔR_BT_/ΔR_RT_ being only 2.1%, 4.1%, and 0.7% for W500-H100, W500-H300, and W1000-H300, respectively (Figure 5B). A more important thing to note is that measurement of ΔR was very consistent and stable at both tested temperatures. These results successfully demonstrate that the developed sensors can stably monitor the ICP over time regardless of the temperature variation within the operating temperature range (23–38 °C).

Next, the linearity of the ICP sensor within the operating pressure range was analyzed by applying incremental cyclic pressures (ΔP = 0 mbar to ΔP = 100 mbar with 10 mbar increment). The operating pressure range was set from ΔP = 0 mbar to ΔP = 100, as it generated sufficient inflation of the fontanelle model (i.e., up to 3.50 mm). As can be seen in Figure 5C, the ΔR in accordance with the pressure change could be measured in real time with high stability. Specifically, the coefficient of variation for W500-H100 was largest with CV = 0.0348 at ΔP = 10 mbar and continued to decrease to reach CV = 0.0135 at ΔP = 100 mbar. The cyclic incremental test also showed that the ICP sensor outputs has extremely high correlation with the applied pressures (Figure 5D). For all three designs, the R^2^ values were higher than 0.9990, with the W500-H100 design showing the highest linear correlation of R^2^ = 0.9999 and W1000-H300 showing the lowest with R^2^ = 0.9992, which still possess a very high linearity. These results successfully demonstrate that the developed ICP sensors can accurately and stably monitor the change of ICP in real time with accuracy over a wide range of operational pressures.

Lastly, the responsiveness of the ICP sensor was analyzed by calculating a time interval to reach from 10% to 90% of the steady state values under the applied pressure condition. The measured response time was 12.11 ± 1.13 (W500-H100), 13.41 ± 1.30 (W500-H300), and 13.69 ± 2.41 (W1000-H300) seconds. These might be slower compared to other previously reported pressure sensors; however, considering that the ICP change is slow and the ICP monitoring requires more of stable measurement over time rather than acute change measurement, the response time of the developed ICP sensor is sufficient, making it suitable for the application. Additionally, it should be noted that the calculated response time includes time required for the fontanelle model to inflate, which means that the actual response time of the sensor itself is shorter.

## 4. Conclusions

In this study, a thin, flexible, and wearable liquid metal-based non-invasive ICP sensor was successfully developed, ruling out complications of conventional ICP measurement methods (e.g., bleeding, infection, etc.). The novel freeze casting method allowed to fabricate the thin ICP sensor encapsulating the Ga structure, which can be directly applied to infant patients like a band-aid. Three different sensor designs were characterized for analyzing design parameters and the W500-H100 design was found to be approximately 1.8 times and 2.4 times more sensitive compared to the other two designs (W500-H300W, W1000-H300). Real time monitoring of electrical resistance change in accordance to periodic pressure cycle showed very stable measurement over time with largest coefficient of variation being only 0.03. In addition, the ICP sensor showed very high linear correlation (R^2^ > 0.9990) for all tested designs within the operating pressure range (ΔP = 0–100 mbar). The results sufficiently demonstrate the use of the developed ICP sensor as an alternative approach for monitoring the ICP of infants in a non-invasive manner at much lower cost.

## Figures and Tables

**Table 1:** ICP sensors with different dimensions for sensitivity characterization.

		ICP Sensor Design		
		W500-H100	W500-H300	W1000-H300
**Gallium structure design**	Width (µm)	500	500	1000
	Height (µm)	100	300	300
	Length (µm)	4.55 × 10^5^	4.55 × 10^5^	4.55 × 10^5^
	Sensor thickness (µm)	500	500	500
